# Development and external validation of a nomogram for predicting postoperative adverse events in elderly patients undergoing lumbar fusion surgery: comparison of three predictive models

**DOI:** 10.1186/s13018-023-04490-1

**Published:** 2024-01-03

**Authors:** Shuai-Kang Wang, Peng Wang, Zhong-En Li, Xiang-Yu Li, Chao Kong, Shi-Bao Lu

**Affiliations:** 1 Department of Orthopedics, Xuanwu Hospital, Capital Medical University, No.45 Changchun Street, Xicheng District, Beijing, China; 2 National Clinical Research Center for Geriatric Diseases, Beijing, China; 3 Department of Orthopedics, Beijing Friendship Hospital, Capital Medical University, Beijing, China

**Keywords:** Elderly patients, Adverse events, Predictive model, Machine learning, Online tool

## Abstract

**Background:**

The burden of lumbar degenerative diseases (LDD) has increased substantially with the unprecedented aging population. Identifying elderly patients with high risk of postoperative adverse events (AEs) and establishing individualized perioperative management is critical to mitigate added costs and optimize cost-effectiveness to the healthcare system. We aimed to develop a predictive tool for AEs in elderly patients with transforaminal lumbar interbody fusion (TLIF), utilizing multivariate logistic regression, single classification and regression tree (hereafter, “classification tree”), and random forest machine learning algorithms.

**Methods:**

This study was a retrospective review of a prospective Geriatric Lumbar Disease Database (age ≥ 65). Our outcome measure was postoperative AEs, including prolonged hospital stays, postoperative complications, readmission, and reoperation within 90 days. Patients were grouped as either having at least one adverse event (AEs group) or not (No-AEs group). Three models for predicting postoperative AEs were developed using training dataset and internal validation using testing dataset. Finally, online tool was developed to assess its validity in the clinical setting (external validation).

**Results:**

The development set included 1025 patients (mean [SD] age, 72.8 [5.6] years; 632 [61.7%] female), and the external validation set included 175 patients (73.2 [5.9] years; 97 [55.4%] female). The predictive ability of our three models was comparable, with no significant differences in AUC (0.73 vs. 0.72 vs. 0.70, respectively). The logistic regression model had a higher net benefit for clinical intervention than the other models. A nomogram based on logistic regression was developed, and the C-index of external validation for AEs was 0.69 (95% CI 0.65–0.76).

**Conclusion:**

The predictive ability of our three models was comparable. Logistic regression model had a higher net benefit for clinical intervention than the other models. Our nomogram and online tool (https://xuanwumodel.shinyapps.io/Model_for_AEs/) could inform physicians about elderly patients with a high risk of AEs within the 90 days after TLIF surgery.

## Introduction

According to the United Nations 2022 Revision of World Population Prospects, the proportion of people over 65 years of age is expected to increase from approximately 9.7% in 2022 to 16.4% in 2050 [1]. The burden of lumbar degenerative diseases (LDD) has increased substantially with the unprecedented aging population. From 2004 to 2015, the volume of elective lumbar fusion procedures for LDD among those over age 65 in the USA increased by 138%, and the costs for elective lumbar fusion increased from $3.7 billion dollars in 2004 to $10.2 billion dollars in 2015 [2]. Transforaminal lumbar interbody fusion (TLIF) was first reported in the early 1980s as a modification of posterior LIF and has become a commonly used surgical procedure for nerve decompression and bone stabilization with excellent and reliable outcomes [3, 4]. Postoperative adverse events (AEs) following lumbar fusion surgery include complications, prolonged hospital stay, and readmission, which increase hospitalization-related expenditures and postoperative dissatisfaction [5, 6]. In previous studies, elderly patients (aged 65 years and older) had more extended hospital stays and about twice the complication rate of younger patients [7, 8]. Identifying elderly patients with high risk of AEs and establishing individualized perioperative management is critical to mitigate added costs and optimize cost-effectiveness to the healthcare system.

Many independent variables are associated with postoperative AEs following lumbar fusion surgery. Variables associated with increased length of stay (LOS) include increased age, morbid obesity, diabetes, opioid use, greater number of comorbid conditions, unemployment, drain use, and blood transfusion [9–11]. Older and non-married patients, those with obesity, positive smoking history, longer procedure times, and emergent cases were significantly more likely to be readmitted for complications or physical rehabilitation [9, 10, 12]. Variables associated with complications include cerebrovascular disease, electrolyte disorders, hemi/paraplegia, mass blood loss, and postoperative delayed ambulation [13–16]. Predictive models are more likely to provide individualized expectations for postoperative outcomes during preoperative consultation than risk factor analysis alone. Furthermore, although postoperative AEs are more common in the elderly population, research regarding developing predictive models for AEs in elderly patients is lacking.

Multivariate logistic regression and machine learning algorithms are now applied widely across medical field to develop predictive models. Compared with models based on machine learning algorithms, logistic regression models have better stability and model interpretability, while machine learning algorithms have advantages in data processing and nonlinear and multivariate forecasting [17]. Here, we sought to develop a predictive tool for postoperative AEs in elderly patients, utilizing multivariate logistic regression, single classification and regression tree (hereafter, “classification tree”), and random forest machine learning algorithms.

## Materials and methods

### Patient population

This study was a retrospective review of a prospective Geriatric Lumbar Disease Database, which includes basic information (including demographic data, medical disease, laboratory test, and medication history), perioperative data, and follow-up results of consecutive patients aged 65 years and older. The Institutional Review Board approved the study (IRB# 2018086). Due to the nature of this retrospective study, the informed consent from patients was waived. We reviewed data of patients who underwent elective fusion surgery for lumbar degenerative disease between August 2018 and October 2022 and were followed up for more than three months postoperatively. Inclusion criteria were as follows: (i) aged 65 years and over; (ii) patients with elective TLIF surgery. Exclusion criteria were as follows: patients with (i) irreversible loss of mobility before admission; (ii) revision surgery; (iii) preexisting spinal fracture, any spinal infection or any malignancy; (iv) incomplete data; and (v) surgery-related complications including incidental durotomy, nerve injury or spinal cord injury.

### Predictive variables

The demographic and clinical data included age, gender, weight, body mass index (BMI), payment type, medical disease (Charlson comorbidity index, cardiovascular disease, diabetes, medication history (glucocorticoid and anticoagulant), osteoporosis, current smoker or drinker, etc.), and laboratory tests (red blood cell count [RBC], hemoglobin, and coagulation function tests indicators). Surgery-related variables included the number of fused segments, estimated blood loss (EBL), operative time, and drainage volume on postoperative day 0 (POD0). Given the importance of early ambulation as a protective factor reported in previous studies, we also include delayed ambulation (bed rest for more than 48 h after surgery) as a predictive variable. For external validation, we reviewed a consecutive cohort of 175 elderly patients who underwent lumbar fusion surgery in another hospital.

### Outcome measure

Postoperative AEs included postoperative complications, prolonged LOS, readmission, and reoperation within 90 days after surgery. We excluded patients with intraoperative complications that were related to the surgical technique of surgeons. Prolonged LOS was defined as postoperative hospital stay greater than the 75th percentile. The indications for readmission included medical complications, reoperation, physical rehabilitation, and other unplanned readmissions. The LOS was recorded routinely by hospital administrative staff who were unaware of the study and extracted from the hospital electronic patient record by a research nurse. Other AEs were recorded by another research nurse using predefined criteria for the presence or absence of complications and readmission according to the clinical record, medication record, and follow-up data. Data were taken from the clinical records made by usual care teams unaware of the study. Patients were grouped as either having at least one adverse event (AEs group) or not (No-AEs group).

### Statistical analysis

All statistical analyses were conducted using R version 4.2.2 (R Foundation for Statistical Computing), and significance level was set at *p* < 0.05 for all tests. Continuous data were expressed as means ± standard deviation (SD) and were compared using the two-tailed Student’s t test or the Mann–Whitney U test. Median (quartile 1, quartile 3) was displayed for not normally distributed data. Categorical variables were expressed as frequencies with percentages and analyzed using Fisher’s exact and chi-square tests, as appropriate. Data from the Geriatric Lumbar Disease Database were partitioned with an 80/20 split for training and test datasets. Logistic regression models were built using the function *glm* of the R package stats. Single classification trees were produced with the *rpart* package and were pruned to a complexity parameter of 0.02. Random forest classifiers were created using the *randomForest* R package *v*4.2.3 [18].

Trained models are validated using the remaining 20% of available data. Operating characteristic curve (ROC) was accomplished using the *proc* and *rocr* packages. Area under the curve (AUC) was calculated by applying the model to the testing set. After selecting the most appropriate model, we compared the predicted with the observed probabilities and drew a calibration curve to assess model calibration. Then, we performed a decision curve analysis (DCA) to evaluate the clinical benefit of our model. The nomogram function of the *RMS* package of the R software was used to generate the nomogram. Finally, online tool based on our model was developed to assess its validity in the clinical setting (external validation).

## Results

### Patient characteristics in the development and validation dataset

The development set included 1025 patients (mean [SD] age, 72.8 [5.6] years; 632 [61.7%] female), and the external validation set included 175 patients (73.2 [5.9] years; 97 [55.4%] female). The perioperative and follow-up data of participants in the development dataset (including the training and testing datasets) and validation dataset are shown in Table 1. There were no significant differences in demographic data among the three groups. More patients with peripheral vascular disease and osteoporosis were in the validation dataset (*p* < 0.01). There were also significant differences among the groups in number of fused segments and intraoperative EBL (*p* < 0.001). The incidence of postoperative AEs was similar among the three groups (*p* = 0.264).

**Table 1:** Perioperative and follow-up data of training, testing, and validation dataset

Variables	Training dataset	Testing dataset	Validation dataset	*P*
	N = 820	N = 205	N = 175	Value
*Demographic data*				
Age (yr)	72.8 ± 5.6	72.7 ± 5.7	73.2 ± 5.9	0.713
Male n (%)	305 (37.2%)	88 (42.9%)	78 (44.6%)	0.096
Weight (kg)	66.9 ± 10.9	67.9 ± 10.9	68.5 ± 11.0	0.164
BMI (kg/m^2^)	25.6 ± 3.7	25.6 ± 3.6	25.8 ± 3.5	0.799
*Medical disease n(%)*				
CCI				0.611
0 or 1	676 (82.4%)	167(81.5%)	149 (85.1%)	
2 or more	144 (17.6%)	38 (18.5%)	26 (14.9%)	
Hypertension	523 (63.8%)	127 (62.0%)	99 (56.6%)	0.200
Coronary heart disease	176 (21.5%)	44 (21.5%)	27 (15.4%)	0.189
Peripheral vascular disease	33 (4.0%)	4 (2.0%)	15 (8.6%)	0.005^*^
Diabetes	263 (32.1%)	74 (36.1%)	45 (25.7%)	0.093
Cerebrovascular disease	80 (9.8%)	21 (10.2%)	15 (8.6%)	0.850
Osteoporosis	96 (11.7%)	24 (11.7%)	40 (22.9%)	0.001^*^
Connective tissue disease	17 (2.1%)	6 (2.9%)	9 (5.1%)	0.071
Smoker	95 (11.6%)	31 (15.1%)	20 (11.4%)	0.364
Drinker	66 (8.0%)	19 (9.3%)	11 (6.3%)	0.563
Peptic ulcer	24 (2.9%)	3 (1.5%)	3 (1.7)	0.375
*Laboratory test*				
Red blood cell count(× 10^12^/L)	4.3 ± 0.5	4.2 ± 0.5	4.2 ± 0.5	0.850
Hemoglobin(g/L)	130.2 ± 14.8	129.7 ± 14.6	130.2 ± 12.9	0.898
INR	0.97 ± 0.08	0.99 ± 0.13	0.95 ± 0.06	0.001^*^
*Medication history*				
Glucocorticoids	11 (1.3%)	5 (2.4%)	3 (1.7%)	0.525
Anticoagulant agent	146 (17.8%)	43 (21.0%)	7 (4.0%)	< 0.001^*^
*Surgery-related data*				
Number of fused segments	2.02 ± 0.97	2.02 ± 0.95	1.64 ± 0.71	< 0.001^*^
Lumbosacral fusion	367 (44.8%)	79 (38.5%)	43 (24.6%)	< 0.001^*^
Intraoperative EBL (ml)	352.1 ± 330.1	352.5 ± 282.8	200.8 ± 190.3	< 0.001^*^
Operative time (min)	210.6 ± 67.3	214.4 ± 64.7	210.9 ± 68.2	0.768
Drainage volume on POD0 (ml)	114.1 ± 94.9	112.3 ± 81.4	106.7 ± 97.4	0.001^*^
*Postoperative outcomes*				
Delayed ambulation	288 (35.1%)	78 (38.0%)	57 (32.6%)	0.533
Postoperative LOS (d)	6 (5,9)	6 (5,10)	6 (5,7)	< 0.001^*^
Prolonged LOS	190 (23.2%)	55 (26.8%)	42 (24.0)	0.547
Total complications	149 (18.2%)	54 (26.3%)	37 (21.1%)	0.030^*^
Medical complications	122 (14.9%)	46 (22.4%)	32 (18.3%)	0.028^*^
Surgery-related complications	27 (3.3%)	3 (1.5%)	5 (2.9%)	0.379
Readmission within 90 days	36 (4.4%)	7 (3.4%)	2 (1.1%)	0.117
AEs	282 (34.4%)	83 (40.5%)	63 (36.0%)	0.264

BMI, Body mass index; CCI, Charlson comorbidity index; INR, International normalized ratio; EBL, Estimated blood loss; LOS, Length of hospital day; POD0, Postoperative day 0; AEs, Adverse events

*Represents for statistically different (*P* < 0.05)

### Univariate risk analysis for postoperative AEs

Univariate analyses revealed that age (*p* < 0.001), BMI (*p* = 0.015), hemoglobin (*p* = 0.03), osteoporosis (*p* = 0.003), INR (*p* = 0.025) number of fused segments (*p* < 0.001), lumbosacral fusion (*p* = 0.014), intraoperative EBL (*p* < 0.001), operative time (*p* < 0.001), delayed ambulation (*p* < 0.001), drainage volume on POD0 (*p* < 0.001), and diabetes (*p* = 0.031) were significantly associated with postoperative AEs. All models were developed with the training dataset and evaluated with the testing and external validation datasets (Table 2).

**Table 2:** Univariate analysis of risk factors for AEs

	Non-AEs group	AEs group	*P*
Variables	N = 660	N = 365	Value
*Demographic data*			
Age (yr)	71.9 ± 5.2	74.4 ± 6.0	< 0.001^*^
Male n (%)	264 (40%)	129 (35.3%)	0.161
Weight (kg)	66.9 ± 10.9	67.7 ± 10.9	0.255
BMI (kg/m^2^)	25.4 ± 3.6	26.0 ± 3.7	0.015^*^
*Medical disease n(%)*			
CCI			0.098
0 or1	553 (83.8%)	290 (79.5%)	
2 or more	107 (16.2%)	75 (20.5%)	
Hypertension	410 (62.1%)	240 (65.8%)	0.276
Coronary heart disease	133 (20.2%)	87 (23.8%)	0.195
Peripheral vascular disease	24 (3.6%)	13 (3.6%)	1.000
Diabetes	201 (30.5%)	136 (37.3%)	0.031^*^
Cerebrovascular disease	64 (9.7%)	37 (10.1%)	0.907
Osteoporosis	46 (7.0%)	49 (13.4%)	0.003^*^
Connective tissue disease	16 (2.4%)	7 (1.9%)	0.761
Smoker	88 (13.3%)	38 (10.4%)	0.206
Drinker	57 (8.6%)	28 (7.7%)	0.676
Peptic ulcer	16 (2.4%)	11 (3.0%)	0.718
*Laboratory test*			
Red blood cell count(× 10^12^/L)	4.3 ± 0.5	4.2 ± 0.5	0.074
Hemoglobin(g/L)	130.9 ± 14.9	128.8 ± 14.4	0.030^*^
INR	0.97 ± 0.08	0.98 ± 0.11	0.025^*^
*Medication history*			
Glucocorticoids	13 (2%)	3 (0.8%)	0.248
Anticoagulant agent	121 (18.3%)	68 (18.6%)	0.973
*Surgery-related data*			
Number of fused segments	1.8 ± 0.80	2.4 ± 1.1	< 0.001^*^
Lumbosacral fusion	268 (40.6%)	178 (48.8%)	< 0.014^*^
Intraoperative EBL (ml)	296.4 ± 276.9	452.8 ± 367.8	< 0.001^*^
Operative time (min)	198.0 ± 59.4	235.3 ± 72.5	< 0.001^*^
Drainage volume on POD0 (ml)	102.5 ± 79.8	134.0 ± 108.7	< 0.001^*^
Delayed ambulation	183 (27.7%)	183 (50.1%)	< 0.001*

BMI, Body mass index; CCI, Charlson comorbidity index; INR, International normalized ratio; EBL, Estimated blood loss; POD0, Postoperative day 0

*Represents for statistically different (*P* < 0.05)

### Logistic regression model

Multivariate analyses revealed that older age (odds ratio [OR] 1.84, *p* < 0.001), higher BMI (OR 1.28, *p* = 0.019), more intraoperative EBL (OR 1.22, *p* = 0.036), longer operative time (OR 1.92, *p* < 0.001), and delayed ambulation (OR 1.88, *p* < 0.001) were independent risk factors for postoperative AEs in elderly patients undergoing lumbar fusion surgery (Table 3).

**Table 3:** Multivariate logistic regression for postoperative AEs

Risk factors	OR (95% CI)	*P*-value
Age (yr)	1.84 (1.42–2.40)	< .001
BMI (kg/m^2^)	1.28 (1.04–1.56)	0.019
Intraoperative EBL (ml)	1.22 (1.01–1.47)	0.036
Operative time (min)	1.92 (1.47–2.51)	< .001
Delayed ambulation	1.88 (1.36–2.61)	< .001

BMI, Body mass index; EBL, estimated blood loss

### Single classification tree model

**Fig. 1:**
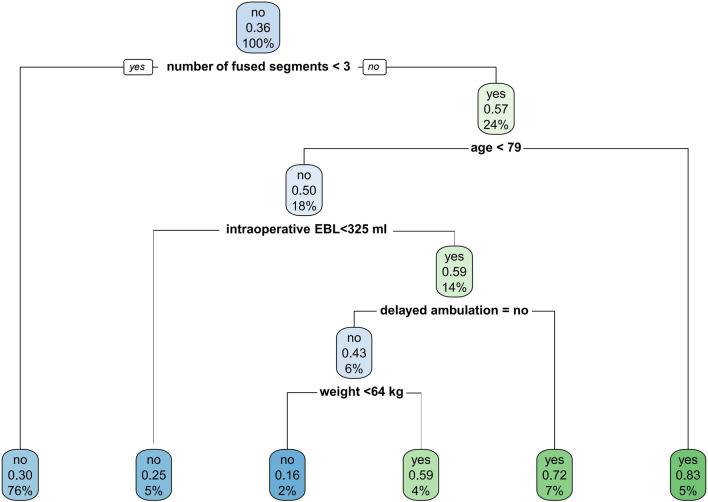
Decision tree for risk of AEs following lumbar fusion surgery

The single classification tree revealed that the number of fused segments ≥ 3, age ≥ 79 years, intraoperative EBL ≥ 325 ml, delayed ambulation, and weight ≥ 64 kg as particularly influential predictors for AEs (Fig. 1).

### Random forest model

**Fig. 2:**
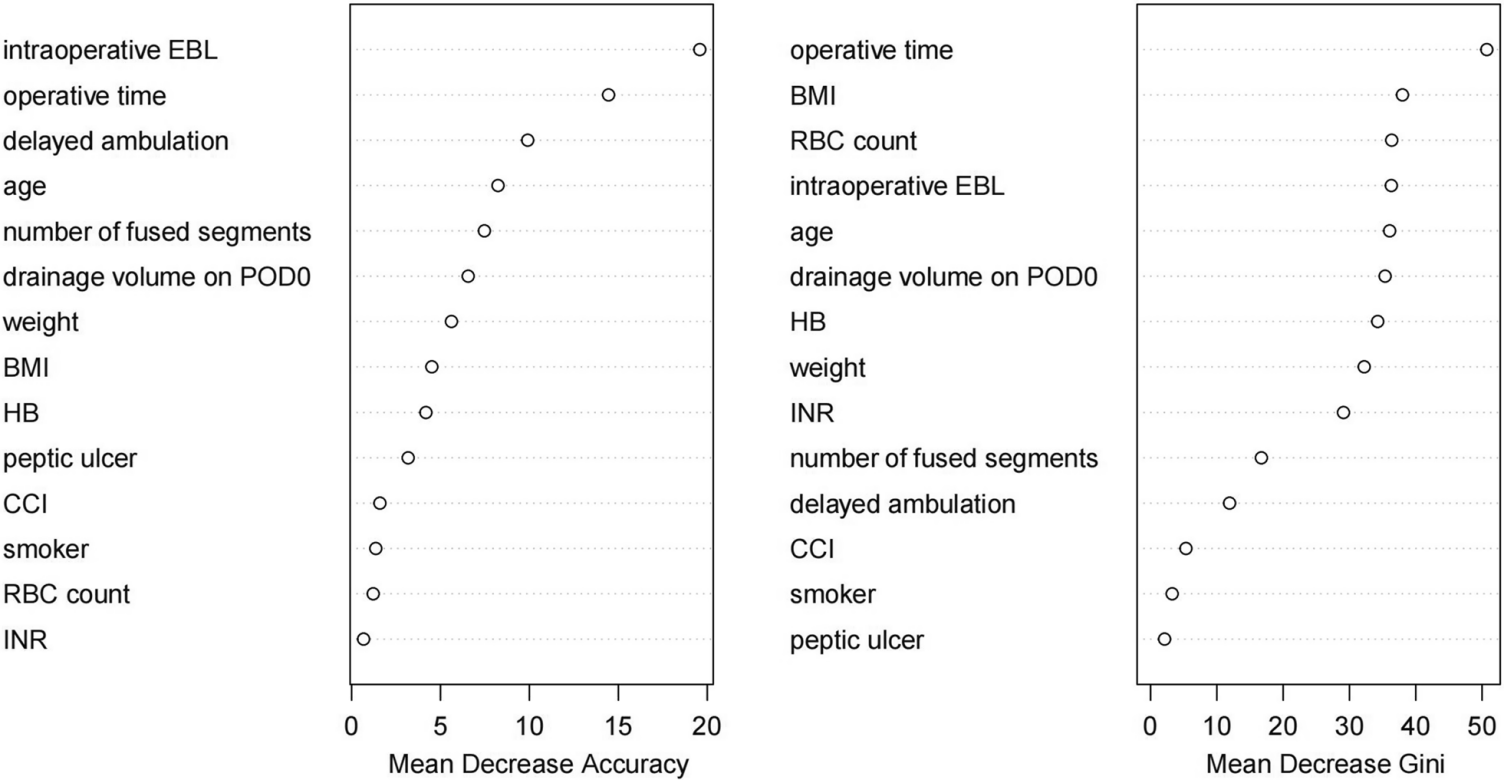
Random forest model variable importance

Recursive feature elimination removed 12 variables from the original set of 26 candidate predictors. A random forest model was developed using the remaining 14 variables. Intraoperative EBL, operative time, delayed ambulation, age, number of fused segments, BMI, and RBC count were the most significant variables in the final model (Fig. 2).

**Fig. 3:**
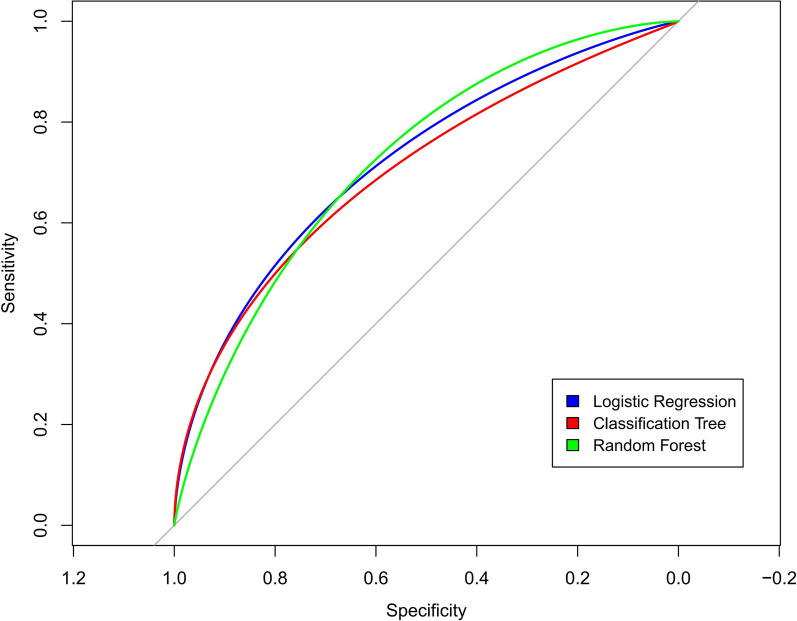
Receiver operating characteristic curves of logistic regression (blue), single classification tree (red), and random forest algorithms (green)

**Fig. 4:**
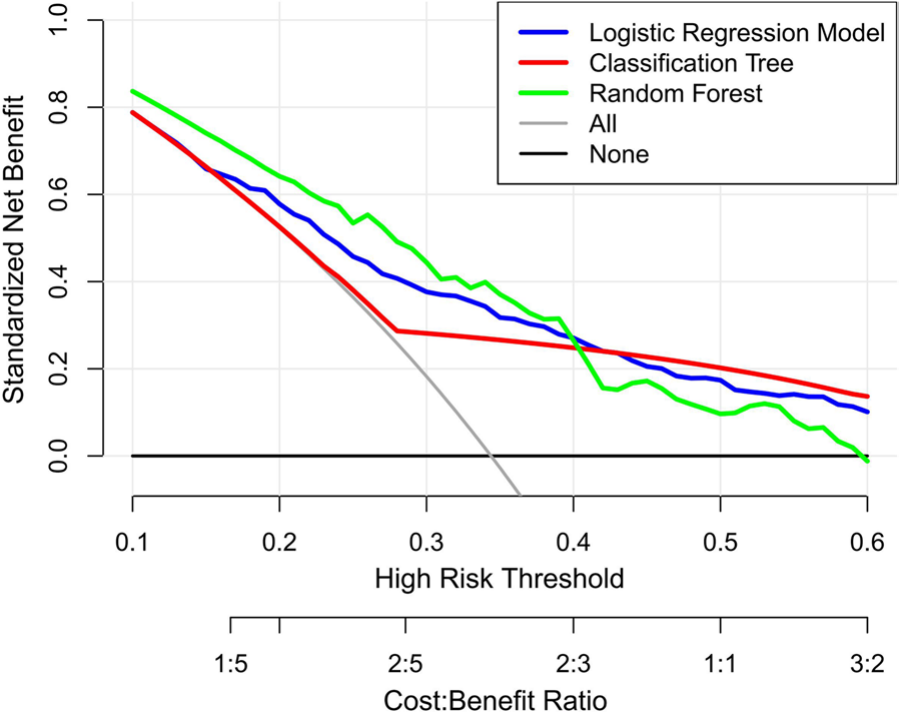
Decision curve analysis comparing the clinical utility of the three models

After the three models were developed using the training dataset, every model performance was validated on a separate cohort of 205 patients in the testing dataset. The logistic regression model AUC was 0.73 vs. 0.72 for the random forest model and 0.70 for the single classification tree model (Fig. 3). DCA was performed to calculate the clinical net benefit of each model, and it revealed that the logistic regression model was more benefit than random forest and single classification tree model in predicting postoperative AEs (Fig. 4).

**Fig. 5:**
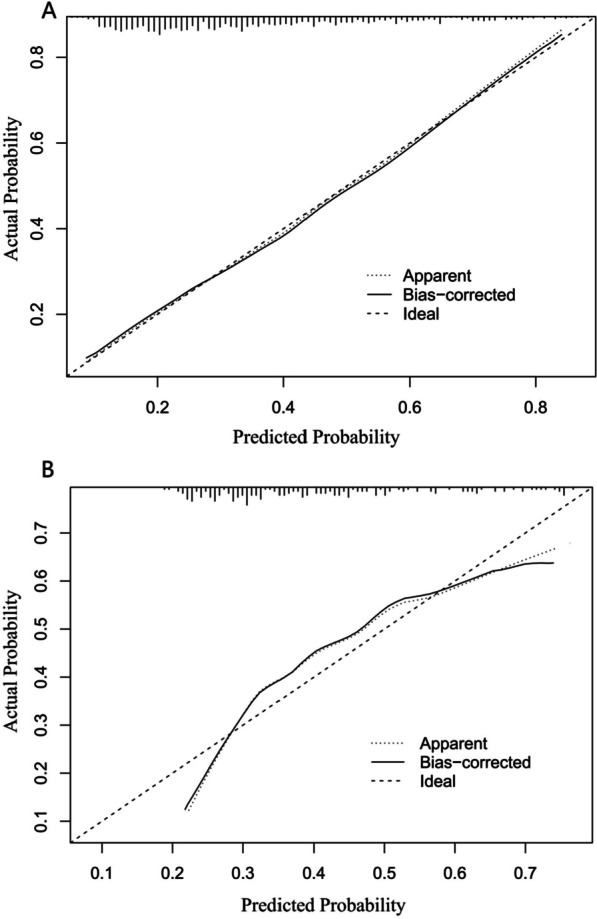
Calibration curve of the training (**A**) and testing (**B**) cohort

**Fig. 6:**
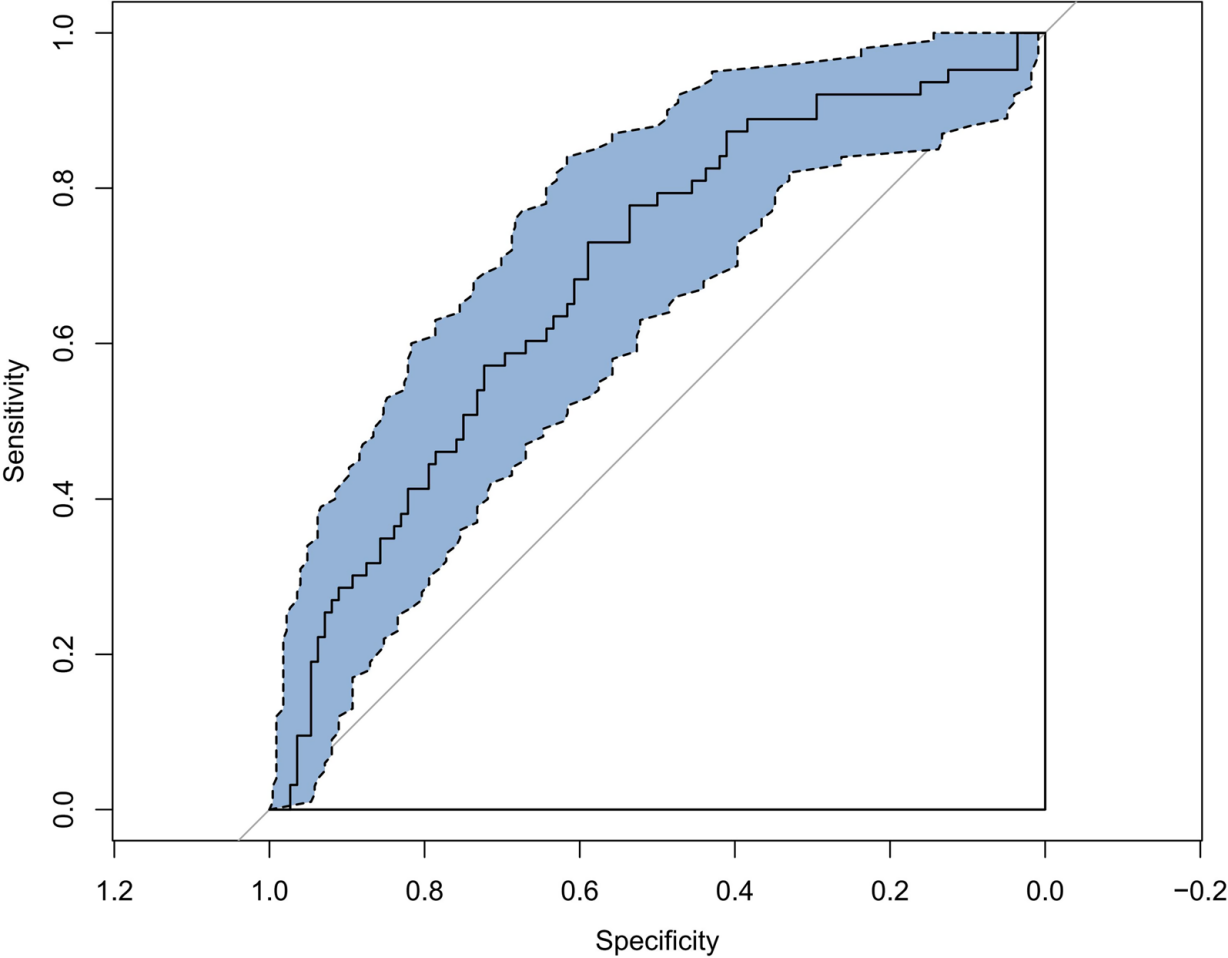
Receiver operating characteristic curve showing sensitivity and specificity of the logistic model for predicting occurrence of any adverse events among the external validation cohort

**Fig. 7:**
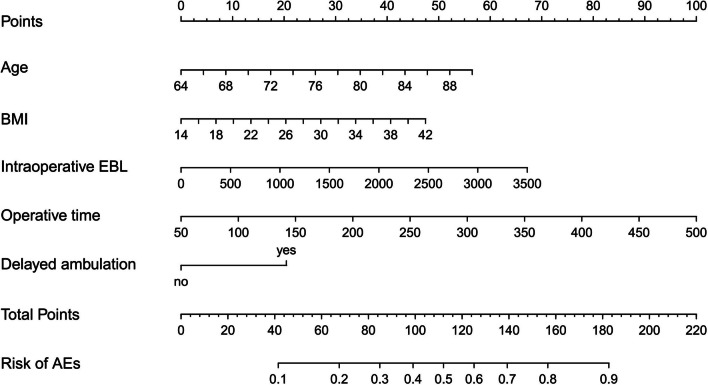
The nomogram based on the multivariate logistic regression model

Finally, we therefore selected the simpler logistic regression model to build our prognostic classifier. The logistic regression model was well-calibrated (observed to expected ratios) in the training and validation cohorts (Fig. 5). The accuracy of the predictive model was 70% (AUC = 0.69) in the sample from an institution that was different and independent from those used for model creation and internal validation (Fig. 6). Then, the logistic regression model data were used to construct a nomogram (Fig. 7).

## Discussion

The hospitalized patient experience has become an area of increased focus for hospitals given the recent coupling of patient satisfaction to reimbursement rates for inpatients [19]. Although previous studies focused on identifying risk factors for complications, reducing LOS and readmission rates are equally important to improve the patient experience and reduce costs [6, 20]. Therefore, postoperative AEs should include complications, readmission, and prolonged LOS after spine surgery. In this study, we developed and validated three models (logistic regression, single classification tree and random forest algorithms) for predicting postoperative AEs following lumbar fusion surgery in elderly patients using a prospective Geriatric Lumbar Disease Database. To our knowledge, this is the first study to develop and externally validate a prediction model for postoperative AEs in elderly patients with high predictive accuracy.

The predictive ability of our three models was comparable, with no significant differences in AUC. Then, we performed a decision curve analysis to determine the clinical usefulness of the three risk stratification models. The logistic regression model had a higher net benefit for clinical intervention than the other models. Our final models (logistic regression model) had good calibration and predictive performance in the internal validation cohort (C-statistics, 0.69–0.76), demonstrating that it can accurately predict potential outcomes in new populations with similar characteristics. Finally, we performed an external validation of the most appropriate model and provided an online user-friendly risk prediction tool at https://xuanwumodel.shinyapps.io/Model_for_AEs/.

Multivariable regression analysis revealed that age, BMI, operative time, intraoperative blood loss, and delayed ambulation were independently associated with postoperative AEs. This finding was in line with other spine surgery literature, which suggested that older age, obesity, and surgical trauma were risk factors for postoperative complications [8, 14, 21]. Intraoperative blood loss was also associated with readmission and prolonged length of hospital stay in previous studies [12, 22]. Our findings suggest that operative time and blood loss were more critical risk factors than the number of surgical segments for AEs in lumbar fusion for degenerative disorders. Preoperative risk of AEs may be modified by intraoperative events, most notably blood loss, which was the most relevant single parameter determining the risk of AEs in elderly patients. For older patients with higher BMI, reducing intraoperative blood loss and operative time can decrease the occurrence rate of adverse events. As an important intervention of enhanced recovery after surgery pathway (ERAS), early ambulation had been demonstrated to be associated with better clinical outcomes [5, 23]. Our study also revealed that delayed ambulation (> 48 h) after surgery was a predictor for postoperative AEs when patients with intraoperative complications were excluded. Therefore, how to improve ERAS compliance of elderly patients is an urgent problem to be solved.

Tree-based machine learning algorithms (including the single classification tree and the random forest approach) were chosen based on many desirable properties for the given binary outcome of having an adverse event or not, including the ability to handle hundreds of variables (both categorical and continuous) and ease of construction [14]. The single classification tree algorithm showed that the number of fused segments was the first discriminator for predicting postoperative AEs. Our findings supported that > 3 fusion segments in lumbar surgery was considered to be long-segment fusion that can cause more extensive surgical trauma. Other discriminators of the present classification tree included age, intraoperative blood loss, delayed ambulation, and weight, similar to our regression analysis and some prior studies [13, 18, 24, 25]. In a retrospective multicenter database analysis, Arora et al. [11] performed a decision tree analysis and found that advanced age, obesity, and greater surgical invasiveness were significant variables increasing the likelihood of readmission and prolonged LOS. To reduce the incidence of postoperative AEs, in older patients (aged 79 years or older) who undergo long-segment fusion, hemostatic agents and minimally invasive surgery should be used to reduce intraoperative bleeding.

Random forest is an algorithm that, in many situations, improves on single classification trees. However, the random forest model exhibited a nonsignificant trend to superior discrimination compared to the classification tree model (classification tree AUC = 0.70 vs. random forest AUC = 0.72). This lack of significant difference is likely due to the small size of our testing dataset, as the method utilized to calculate AUC confidence intervals dependent on sample size. Similar to the other two models, the most important variables in the random forest model were operative time, intraoperative blood loss, BMI, age, and delayed ambulation. Preoperative RBC count and drainage volume on POD0 were relatively important predictors for postoperative AEs within 90 days of surgery. These two variables correlate highly with postoperative RBC count and hemoglobin, which could affect discharge planning. This conjecture, however, will require further study.

Previous spinal surgery studies focused solely on comparing different machine learning and regression approaches for postoperative AEs in patients with spine deformity and involved only internal validation of the developed prediction models [13, 25]. Yagi et al. and Passias et al. demonstrated the efficacy of classification trees in preoperative screening and risk stratification of patients likely to have major complications following corrective spine surgery and cervical deformity surgery, respectively [13, 26]. In a retrospective study of 37,852 patients, Jain et al. developed nine models to assess risks of discharge-to-facility, 90-day readmissions, and major medical complications after long-segment lumbar spine fusion with moderate sensitivity and specificity. The authors found that logistic regression models modestly outperformed random forest and elastic net models [25]. Although many predictive models for spinal disorders had been developed, that as yet we have to pay careful attention in deciding which tools to use depending on the outcomes and the setting of interest. Despite the considerable rate of AEs in elderly patients undergoing lumbar fusion surgery, there are few quantitative tools to predict AEs in elderly patients. The present study offers a novel contribution to the field by assessing and comparing the performance of logistic regression models versus tree-based algorithms and validating them using external data.

This study had several limitations. First, our models were created using retrospective data and are relevant to context of the current standard of care. Prospective multicenter studies can guarantee the sustained effectiveness of the model while ensuring a large sample size. However, our retrospective and uncontrolled data theoretically lends to greater generalizability across institutions and surgical teams. Second, there might be significant predictors of outcomes that are not available in our data set, such as insurance status, dependency status, income levels, and details of spinal pathology. In the present study, we found many non-modifiable preoperative characteristics and intraoperative variables were significantly risk factors for postoperative AEs. Of note, our primary goal was to predict the absolute risk of AEs following surgery and not to identify modifiable factors. Third, given that postoperative complications mainly occurred within the first three months after surgery, we included only patients who were followed up for three months or more [27, 28]. Finally, as we move toward value-based care and shared decision-making, there is an increasing need to collect and use patients-reported outcomes not just in research settings, but also in routine clinical care or quality improvement activities. Thus, our future research will develop models for postoperative minimal clinically important difference and satisfaction in elderly patients undergoing lumbar fusion surgery with a long-term follow-up.

## Conclusions

This investigation produced three predictive models for postoperative adverse events in elderly patients undergoing lumbar fusion surgery. The predictive ability of our three models was comparable. Logistic regression model had a higher net benefit for clinical intervention than the other models. An online dynamic nomogram calculator was established based on the final logistic regression model. Our predictive tool could inform physicians about elderly patients with a high risk of AEs within the 90 days after surgery.

## Data Availability

The underlying data supporting the results of this study could be obtained by contacting the corresponding author.

## Abbreviations

LDD: Lumbar degenerative diseases
TLIF: Transforaminal lumbar interbody fusion
AEs: Adverse events
LOS: Length of hospital stay
BMI: Body mass index
RBC: Red blood cell count
EBL: Estimated blood loss
POD: Postoperative day
ROC: Operating characteristic curve
AUC: Area under the curve
DCA: Decision curve analysis
ERAS: Enhanced recovery after surgery
